# Supplementary material for: Robotic Care Equipment Improves Communication between Care Recipient and Caregiver in a Nursing Home as Revealed by Gaze Analysis: A Case Study
Source: Int J Environ Res Public Health. 2024 Feb 22;21(3):250. doi: 10.3390/ijerph21030250 (PMC10970074; doi:10.3390/ijerph21030250)
Supplement: Supplementary file 1 [file ijerph-21-00250-s001.zip › ijerph-2833599-SI.pdf]

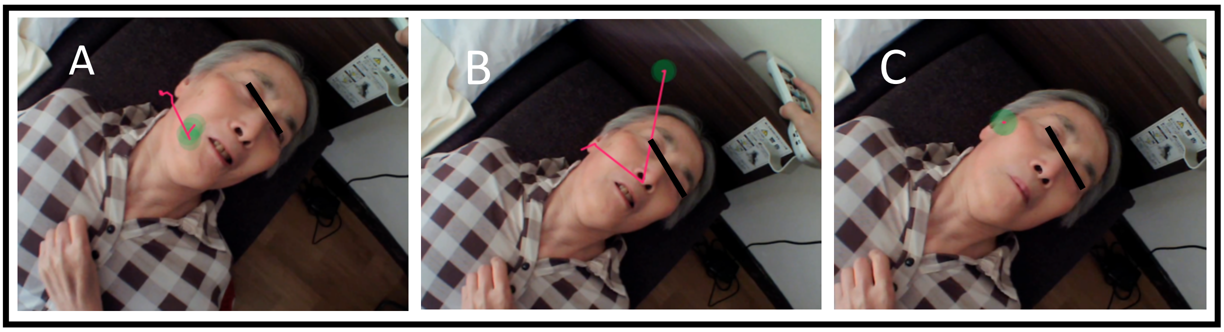

Figure S1. Facial expression of the care recipient.

Smiling face (A, B) and usual expression (C) of the care recipient during Resyone separation.
